# Glucagon, GLP-1 and Thermogenesis

**DOI:** 10.3390/ijms20143445

**Published:** 2019-07-13

**Authors:** Ismael González-García, Edward Milbank, Carlos Diéguez, Miguel López, Cristina Contreras

**Affiliations:** 1 Institute for Diabetes and Obesity, Helmholtz Diabetes Center, Helmholtz Zentrum München, German Research Center for Environmental Health (GmbH), 85764 Neuherberg, Germany; 2 Center for Research in Molecular Medicine and Chronic Diseases (CIMUS), University of Santiago de Compostela-Instituto de Investigación Sanitaria, 15782 Santiago de Compostela, Spain; 3 Centro de Investigación Biomédica en Red, Fisiopatología de la Obesidad y Nutrición (CIBERobn), 15706 Santiago de Compostela, Spain; 4 Department of Physiology, Pharmacy School, Complutense University of Madrid, 28040 Madrid, Spain

**Keywords:** glucagon, GLP1, thermogenesis, brown adipose tissue, browning, hypothalamic control of energy balance

## Abstract

Brown adipose tissue (BAT) thermogenesis is a conserved mechanism to maintain body temperature in mammals. However, since BAT contribution to energy expenditure can represent a relevant modulator of metabolic homeostasis, many studies have focused on the nervous system and endocrine factors that control the activity of this tissue. There is long-established evidence that the counter-regulatory hormone glucagon negatively influences energy balance, enhances satiety, and increases energy expenditure. Despite compelling evidence showing that glucagon has direct action on BAT thermogenesis, recent findings are questioning this conventional attribute of glucagon action. Glucagon like peptide-1 (GLP-1) is an incretin secreted by the intestinal tract which strongly decreases feeding, and, furthermore, improves metabolic parameters associated with obesity and diabetes. Therefore, GLP-1 receptors (GLP-1-R) have emerged as a promising target in the treatment of metabolic disorders. In this short review, we will summarize the latest evidence in this regard, as well as the current therapeutic glucagon- and GLP-1-based approaches to treating obesity.

## 1. Introduction

One of the most important aspects in animal physiology is the proper regulation of energy balance. A high number of homeostatic mechanisms fall under this pure thermodynamic concept when considering the animal as a whole. Essentially, these mechanisms will focus on the two components of energy balance: regulation of food intake and energy expenditure [1,2]. Energy expenditure can be divided into different forms: basal metabolism, physical activity, and adaptive thermogenesis. Mainly due to the recent advances and discoveries in the field demonstrating its importance in humans [3,4,5,6], adaptive thermogenesis is not only considered an essential mechanism for the maintenance of body temperature, but also for whole body metabolic homeostasis [7].

Adaptive thermogenesis is an essential mechanism in warm-blooded animals, occurring when ambient temperature is lower than thermoneutrality to maintain the core internal temperature (above this point, physiological basal thermogenesis is sufficient to maintain body temperature without activating other homeostatic mechanisms) [8]. Under this threshold, heat-saving mechanisms are activated, such as cutaneous vasoconstriction, piloerection, and the adoption of spherical postures and the reduction of mobility. When these mechanisms are insufficient to maintain body temperature within a physiological range, facultative thermogenesis is activated through two processes: (i) shivering and (ii) non-shivering that takes place in the brown adipose tissue (BAT). In the BAT, the dissipation of the energy through heat production is mediated by the decoupling of mitochondrial respiration by the uncoupling protein 1 (UCP1) [7,8]. Although brown adipocytes possess receptors for peripheral signals and are able to respond to direct modulation, the physiological regulation of BAT is mainly exerted by the central nervous system. Different signals will act on the pre-optic area (POA), as well as the different hypothalamic nuclei to modulate BAT thermogenesis. A deeper review in this regard can be found in references [7,9,10,11].

Among those factors, glucagon and glucagon-like peptide 1 (GLP-1) have been associated in recent years with BAT thermogenesis, and they will be the main topic of this review. Both glucagon and GLP-1 are products of the proglucagon peptide encoded by the *preproglucagon* gene (*Gcg*). Proglucagon is differentially processed by the predominant expressed prohormone convertases (i.e., prohormone convertase 1/3 and prohormone convertase 2) [12]. The main source of glucagon is the pancreatic α-cell, while intestinal L-cells and neurons in the nucleus of the solitary tract are the principal producers of GLP-1. Physiologically, glucagon’s main action is related to glucose homeostasis, where glucagon stimulation of hepatic glucose production by increasing glycogenolysis and gluconeogenesis, and simultaneously inhibiting glycogen synthesis, serves to restore glucose levels in hypoglycemic states. Due to this action, glucagon is defined as a counter-regulatory hormone. Metabolic actions of glucagon are exerted through a unique receptor which is mainly expressed in liver, but can also be found in brain, heart, kidney, gastrointestinal tract, white fat, and BAT [13,14]. On the other hand, GLP-1 is defined as an incretin hormone, secreted following food intake, and its action (i) stimulates insulin release, (ii) inhibits glucagon secretion, (iii) increases insulin sensitivity in skeletal muscle, and (iv) decreases hepatic gluconeogenesis [12]. Moreover, GLP-1 has additional metabolic actions such as cardioprotective effects and body weight reduction [15], albeit at a higher dose than that needed to exert its effects on glucose homeostasis.

## 2. Glucagon and Thermogenesis

As mentioned, beyond its main action in glucose homeostasis, glucagon has additional metabolic roles. There is long-standing evidence that glucagon decreases food intake [16,17,18,19]. The underlying mechanism is based on an increase in satiety inducing a decrease in meal size without affecting the meal frequency [20,21]. Although this effect on eating behavior influences energy balance, it is also known that glucagon modulates energy expenditure. The first evidence that pointed to a direct action of glucagon in energy expenditure came from two works published in 1960. These two elegant studies were able to demonstrate that the subcutaneous administration of glucagon induced an increase in energy expenditure in rats [22], and, together with this, when the pairfed animals were treated with glucagon, they had an additional weight loss that would imply a glucagon effect on energy expenditure [23]. Interestingly, later studies have demonstrated that the weight loss observed after glucagon treatment in Zucker rats is not accompanied by a concomitant decrease in food intake [24]. In humans, similar data support the glucagon-induced energy expenditure hypothesis [25].

**Figure 1:**
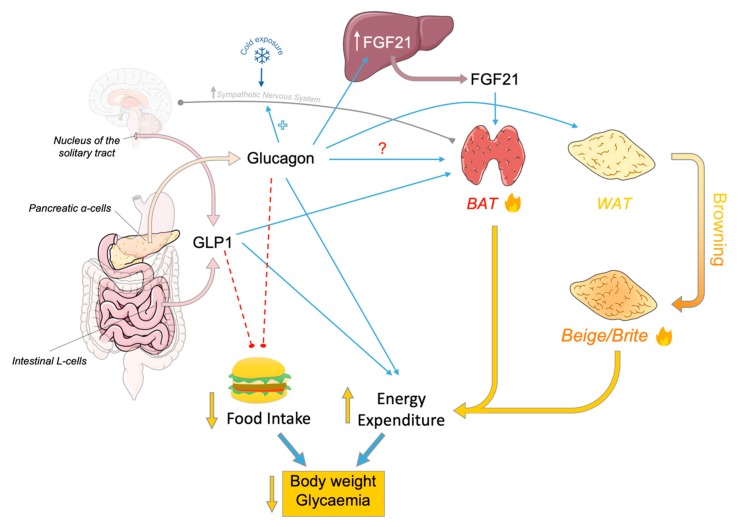
L cells from the digestive tract and nucleus of the solitary tract release glucagon-like peptide-1 (GLP-1) which inhibits feeding, as well as favors thermogenesis in brown adipose tissue (BAT) leading to augmented energy expenditure. Pancreatic alpha-cells release glucagon that apparently stimulates thermogenesis in BAT through favoring cold acclimation and also fibroblast grow factor 21 (FGF21) release in the liver which acts activating thermogenic process in BAT. Furthermore, glucagon may directly stimulate BAT and browning of white adipose tissue (WAT). GLP-1-dependent inhibition of food intake and glucagon-dependent augmentation of energy expenditure have demonstrated beneficial effects on type II diabetes and obese patients leading to reduction of body weight, glycaemia and other metabolic disorders. Direct positive effect (blue arrows), direct negative effect (red dashed lines) and sympathetic efferent projection (grey line). Figure made with Servier Medical Art resources.

As mentioned at the beginning, one of the main components of energy expenditure is brown fat thermogenesis, and several studies in the literature point out that glucagon plays a role in this regard. The first evidence that suggested a possible direct action of glucagon in brown adipose tissue was published by Cliffe D. Joel in 1966, in which it was shown that direct administration of glucagon in BAT slices increased both oxygen consumption and the release of free fatty acids [26]. Thirteen years later, these findings were confirmed by showing that the stimulation of brown fat cells by glucagon could induce heat generation. Moreover, it was also shown that while heat acclimation reduced the response of the brown adipocytes to glucagon, cold acclimation did not modify it [27]. However, when later studies were performed in vivo, a potentiation by cold acclimation was reported. Thus, glucagon induced increased (i) oxygen consumption, (ii) body temperature, and (iii) BAT temperature in rats, and these thermogenic responses to glucagon were improved by cold temperatures while being reduced above thermoneutrality if compared with warm controls [28]. Accordingly, cold exposure and acclimation are associated with an increase in glucagon plasma levels in both rats and humans [29,30,31], and with an increase in BAT glucagon content [32], suggesting that glucagon may play a role in the metabolic adaptation to cold exposure (Figure 1).

In addition to the physiological aspects of glucagon, numerous therapeutic agents based on this pancreatic hormone have recently been developed. The most relevant results were obtained jointly by the Di Marchi and Tschöp groups [33,34], showing promising results in the management of metabolic pathologies such as obesity and diabetes. In these studies, single-molecule, dual- (e.g., Glucagon-GLP1) and tri-agonists (e.g., glucagon-GLP1-GIP) were generated, using glucagon as a template molecule in which chemical variations were introduced. Agonists induced a massive decrease in body weight in diet-induced obese mice [33,35,36,37], and the magnitude of weight loss was actually dependent on the glucagon activity. Interestingly, part of the mechanism of action of these conjugates is based on the increase in energy expenditure induced by the glucagon component. More specifically, those studies have shown how pharmacological treatments could increase the expression of BAT thermogenic markers [35].

Nevertheless, recent genetic models have allowed a deeper understanding of the glucagon action in BAT thermogenesis. Firstly, upon cold exposure, proglucagon-derived peptide knock-out mice (*Gcg*KO mice) respond with a higher decrease in body temperature and an ameliorated increase in oxygen consumption upon cold exposure when KO animals are compared with control wild types [38]. Thermogenic markers in the BAT (e.g., *Ucp1*, *Dio2*, and *Ppargc1a*) were less expressed in GCGKO mice under cold exposure, while UCP1 was also reduced in ambient conditions. Interestingly, BAT stimulation with β3 adrenergic receptor agonist treatment blunted the increase in oxygen consumption observed in *Gcg*KO mice. Moreover, supplementation with glucagon was shown to improve the response to the adrenergic agonist in those *Gcg*KO mice. The last study regarding this was recently published by Drucker’s lab, and pointed out the independence of BAT glucagon receptor (GCGR) in the regulation of energy (food intake, energy expenditure, body composition) metabolism [39]. In fact, the administration of glucagon induced oxygen consumption to a similar extent in *Gcgr*BAT+/+ vs. *Gcgr*BAT−/− and *Ucp1*+/+ vs. *Ucp1*−/− mice, indicating that glucagon increases energy expenditure through a mechanism independent of GCGR and UCP1. Interestingly, these results confirm the preliminary findings reported by Nedergaard’s lab more than twenty years ago. Using hamsters (model displaying glucagon-insensitive brown fat cells), they showed that the injection of glucagon could induce a thermogenic response, indicating that the thermogenic effect appeared not to be due to a direct effect of glucagon on brown fat cells [40].

Several lines of evidence support the idea that the mechanism through which glucagon increases energy expenditure involves fibroblast growth factor 21 (FGF21) [39,41]. Produced and secreted by the liver, FGF21 is a member of the FGF superfamily with diverse metabolic actions. FGF21 decreases glycemia and lipidemia, as well as inducing weight loss by increasing energy expenditure through the activation of BAT thermogenesis and WAT browning [42]. Regarding its link with the pancreatic hormone, it has been reported that glucagon could partially control glucose, energy, and lipid metabolism at least in part via FGF21-dependent pathways [41]. It is known that circulating FGF21 levels were rapidly increased following glucagon administration, while the glucagon-induced elevation in oxygen consumption was partially blunted in *Fgf21*−/− mice. Other observations have suggested that glucagon administration may increase oxygen consumption in the liver, adipose tissue, and possibly other tissues [41]. Nevertheless, those findings about the incomplete abolition of the glucagon response in *Fgf21* null mice open doors to additional GCGR-dependent mechanisms, which could contribute to the acute GCG-dependent enhancement of energy expenditure [43].

Some evidence has also pointed to the possible thermogenic action of glucagon in humans. The effect of hyperglucagonemia on resting metabolic rate (RMR) has been studied in subjects by indirect calorimetry, indicating that there was a significant increase in RMR during glucagon infusion [25]. Glucagon-stimulated thermogenesis was also investigated by measuring energy expenditure (EE) through indirect calorimetry, suggesting that hyperglucagonemia induced an increase of basal EE. Interestingly, the thermogenic effect of glucagon was completely blunted when insulin levels were increased seven-fold over the basal concentration [44]. However, some recent studies have raised some contradictory results. For instance, it was described that glucagon infusion did not increase neck temperature or BAT glucose uptake in healthy male humans [45]. Additionally, contrarily to rodents, its effect in humans does not appear to be mediated by FGF21 [45]. A recent study has raised contradictory results by describing that glucagon infusion did not increase any neck temperature nor BAT glucose uptake in healthy male humans [45], however they also showed that glucagon induced an increase in energy expenditure of a similar magnitude to the one observed with cold activation, confirming the thermogenic effect of glucagon [45]. The insights of these conflicting views are currently not clearly decipherable, and reflect the complex nature of the physiological and signaling mechanisms involved in glucagon action. In conclusion, whether glucagon has a direct action on BAT thermogenesis requires further exploration.

On the other hand, it has been described that glucagon could be involved in the browning of white adipose tissue (WAT). The browning phenomenon consists of the appearance of beige or *brite* adipocytes (brown in white) in the WAT deposits, for example, after cold stimulation or by adrenergic agonists [46]. These beige adipocytes express thermogenic markers and have intermediate anatomical—functional characteristics between brown and white adipocytes [47]. In this regard, it has been studied whether glucagon was required for WAT browning under cold exposure. *Gcg*KO mice presented modest increases of browning at room temperature while displaying a blunted induction of *Ucp1*, *Cidea*, and *Ffg21* expression in inguinal WAT (iWAT) following cold exposure. This effect was also reported in BAT, but with less evidence than in iWAT. Interestingly, glucagon treatment increased the expression of thermogenic genes in both iWAT and BAT of wild type mice (Figure 1). Taken together, these data indicate that glucagon and its signaling pathway could be involved in cold-induced browning of WAT and browning of WAT itself [48].

## 3. Effects of GLP-1 on the Energy Balance

As previously introduced, GLP-1 is a peptide released in response to nutrient ingestion by the L-cells of the gastrointestinal tract, mainly located in the distal small intestine and colon [49,50], and by the nucleus of the solitary tract [51]. GLP-1 action on energy metabolism is mediated by its binding to GLP-1 receptors (GLP-1R), located in both peripheral tissues (as β-pancreatic cells and vagal afferents fibers) and central nervous system (CNS).

It has been demonstrated that endogenous GLP-1 plays a physiological role in suppressing food intake via a paracrine effects [52]. Postprandial GLP-1 binds to GLP-1R expressed in the vagal afferent neurons, inducing firing of cultured vagal afferent neurons [53,54]. Furthermore, the vagotomy surgical procedure blocks the suppressive effects of intraperitoneal (IP) GLP-1 [55,56], suggesting a parasympathetic-nervous-system-dependent effect of GLP-1. It has also been demonstrated that body weight loss in patients undergoing bariatric surgery is partly dependent on GLP-1 action [57,58]. Besides feeding reduction, GLP-1 also (i) facilitates insulin secretion in the pancreas [59,60,61], (ii) decreases hepatic glucose output by gluconeogenesis inhibition [62,63,64,65], (iii) reduces insulin resistance in skeletal muscle [66,67], (iv) slows gastric emptying [68], (v) stimulates β-cell proliferation, and (vi) increases thermogenesis. Altogether, these actions result in beneficial effects on energy balance, reducing body weight and improving metabolic syndrome. Thus, GLP-1 receptor analogs are nowadays recognized as an interesting targets in the treatment of obesity and diabetes.

### Central Effects of GLP-1

The first evidence relating the central effects of GLP-1 on energy balance was related to its ability to reduce food intake [55,56,58,69,70,71,72,73,74], resulting in body weight reduction (Figure 1).

As GLP-1 is quickly degraded by the enzyme dipeptidyl-peptidase IV (DPP-IV) in circulation, the ability of GLP-1 to reach the brain in relevant concentrations remains arguable. Therefore, to circumvent this limitation, numerous studies are using GLP-1 analogues with higher half-life or exogenous GLP-1 infusion in rodent models to elucidate GLP-1 central effects. Consequently, GLP-1 central bio-availability remains unclear; some studies have shown that circulating GLP-1 could reach all brain regions due to its ability to cross the blood brain barrier (BBB), while others studies have limited GLP-1 location to restricted areas with incomplete or leaky BBB, such as the area postrema (AP) and the subfornical organ [51,58,75,76,77]. However, it was shown that when the AP was damaged, the effect of an infusion of GLP-1 in the hepato-portal vein was blocked [58,78]. Other studies have claimed that peripheral GLP-1 might bind to GLP-1R on the vagal afferent neurons, mainly located in portal circulation, and generate electrical signals to the brain, as was demonstrated in vagotomized rodent models in which GLP-1 did not reduce feeding [58,79,80]. Some evidence has demonstrated that GLP-1-induced food intake decreases depending on vagal innervation only when GLP-1 is administrated intraperitoneally but not intravenously [81]. Conversely, another study, carried out in humans, demonstrated that the effect of intravenously injected GLP-1 on feeding was dependent on the vagal nerve [82]. Interestingly, it has been shown that rats consuming a 3 g meal displayed significant elevations in plasma GLP-1 within 5–6 min only in the hepatoportal vein and not in the vena cava [78], suggesting that, under physiological conditions, GLP-1R within the CNS is only reached by brain-derived GLP-1, since GLP-1 is also expressed by a discrete population of caudal brainstem neurons localized to neurons within the caudal (visceral) nucleus of the solitary tract (cNST) and adjacent medullary reticular formation in rodents and primates, including humans [83,84,85,86].

Intracerebroventricular (ICV) administrations of several peptides derived from the proglucagon gene, namely proglucagon-derived peptides (PDP), as glucagon, GLP-1, and oxyntomodulin (OXM) have been demonstrated not only to reduce feeding, but also to promote BAT thermogenesis that is correlated with sympathetic fiber activity innervating brown fat (Figure 1). However, *Glp-1R* KO mice displayed a normal response to cold exposure, suggesting that endogenous GLP-1R is not essential for a normal thermogenic response [87]. Accordingly, the central infusion of exendin-4, a GLP-1R agonist, increased the sympathetic tone of the fibers innervating the BAT, resulting in activation of thermogenesis in both lean and obese mice. The high activity of brown fat induced the clearance of triglycerides and glucose from plasma, reducing white fat depots and favoring body weight loss [88]. Evidence in rodents demonstrates that GLP-1 also regulates body temperature [89] and activates pathways associated with increased thermogenic markers in beige and brown adipocytes [87].

Other reports suggest that central GLP-1R mediates adipocyte metabolism and inhibits triglyceride storage in white fat depots, indicating that central GLP-1R may promote a catabolic state in adipose tissue through sympathetic activation [90]. *Preproglucagon* gene expression in the brainstem is positively correlated with the degree of obesity induced by a high fat diet [91].

Recent studies have described some underlying neuronal pathways mediating PDP-induced thermogenesis. As reported, GLP-1R is highly expressed in the hypothalamus, and is mainly associated with the control of energy balance [92]. Moreover, GLP-1R has been shown to be expressed in POMC neurons within the arcuate nucleus of the hypothalamus (ARC) [93], suggesting that melanocortin pathways may play a key role in the central control of thermogenesis, as happens with the feeding process [94]. Furthermore, GLP-1R is expressed in other hypothalamic nuclei with functions closely related to the control of thermogenesis, such as the dorsomedial nucleus of the hypothalamus (DMH) and medial preoptic area (MPOA) [95], nuclei that also receive projections from brainstem proglucagon neurons [96].

In recent years, new hypothalamic pathways have been reported as targets of GLP-1. For instance, central administration of liraglutide, an analog with a long half-life, activates GLP-1R in the ventromedial nucleus of the hypothalamus (VMH), triggering AMP-activated protein kinase (AMPK) inhibition and leading to sympathetic-induced BAT thermogenesis and browning of WAT, as well as reduced food intake [97]. The AMPK-VMH-SNS-BAT/WAT is a well described hypothalamic pathway activated by several peripheral signals [98,99,100,101], however, this study described for the first time a link between GLP-1R activation and this canonical route.

Besides the hypothalamus, GLP-1R is also located in extrahypothalamic areas, such as the inferior olive (IO) and solitary tract [92,102], that have been previously associated with thermogenesis, suggesting their implication in the central effects of GLP-1 on the thermogenesis and energy expenditure.

On the other hand, new evidence has demonstrated that GLP-1 agonism also plays a key role in glial cells, thus, astrocytes in the NTS (solitary nucleus) could regulate the central effects of GLP-1 controlling the energy balance, as demonstrated by the pharmacological inhibition of NTS astrocytes attenuating the anorectic and body-weight-suppressive effects on intra-NTS GLP-1R activation [103].

Furthermore, some studies have demonstrated that the immune system plays an important role in the GLP-1 effect on energy homeostasis. Thus, adipose-resident invariant natural killer T (iNKT) cells are one such innate immune cell type with an important role in weight and glycemic control, which are present at high levels in adipose tissue [104]. The ability of GLP-1 to act directly on iNKT cell has been demonstrated, since iNKT cell lines for human express GLP-1R which stimulation results in augmented cAMP levels [105]. Interestingly, iNKT cells have anti-inflammatory functions, and their levels are significantly reduced during obesity [106,107]. Thus, recent data demonstrate that iNKT cells contribute to GLP-1-associated weight loss effects by favoring thermogenesis in BAT [108,109].

## 4. New Therapeutic Approaches of Glucagon and GLP-1 Against Obesity

The ability of GLP-1R agonism to reduce body weight and improve glucose metabolism has been well confirmed in numerous preclinical and clinical studies. However, due to the short half-life of GLP-1 (few minutes) when administrated to humans (intravenous or subcutaneous injections) [110], GLP-1 analogues have been developed with the aim to improve their bioavailability and effects. These analogues have attracted the interest of pharmaceutical companies due to their well demonstrated beneficial effects on obesity and diabetes. Several GLP-1 receptor agonists are now approved for therapeutic use as second-line therapy in the treatment of type 2 diabetes. These include exenatide (*Byetta*; AstraZeneca, Cambridge, UK), lixisenatide (*Lyxumia*; Sanofi, Paris, France), liraglutide (*Victoza*; Novo Nordisk, Copenhagen, Denmark), dulaglutide (*Trulicity*; Eli Lilly & Co., Indianapolis, IN, USA), albiglutide (*Tanzeum*; GlaskoSmithKline, Middlesex, UL, USA), semaglutide (Novonordisk, Copenhagen, Denmark), and exenatide (AstraZeneca, Cambridge, UK) [111]. Generally, their effects have been demonstrated in clinical trials in overweight and obese populations, which usually present other risk factors such as hypertension prediabetes/type 2 diabetes, dyslipidemia, and moderate or severe obstructive sleep apnea. [112,113]. Besides weight reduction and a hypoglycemic effect, GLP-1 analogues have also demonstrated beneficial effects on the cardiovascular, nervous, and renal systems, independently of weight loss [114].

The use of agonists of GLP-1R has been demonstrated to be a good therapeutic approach against obesity and type 2 diabetes, by their ability to reach the CNS and modulate metabolic signals, converging in a common molecular mechanism. Therefore, the idea of developing unimolecular multi-agonists has emerged as a new therapeutic strategy [115]. These molecular conjugates have the ability to activate several receptors, so a single molecule displays dual activity and complementary molecular mechanisms, minimizing adverse side effects. This involves using a unique molecule with the ability to activate two molecular mechanisms with a unique pharmacokinetic profile, instead of two. The first developed molecule aimed to simultaneously activate glucagon and GLP-1 receptors for the treatment of obesity and glucose intolerance [35], improving body weight and glycemic control in a similar manner to exendin-4, a GLP-1 analogue [116]. The hypothesis underlying this mechanism was that the hypoglycemic effects of GLP-1 could firstly counteract the hyperglycemic potential of glucagon, while the anorectic effect of central GLP-1 agonism would complement glucagon’s own anorectic, lipolytic, and thermogenic properties to maximize body weight loss. Interestingly, chronic administration of the glucagon/GLP-1 agonist in diet-induced obese (DIO) mice was able to reverse obesity, to improve hepatic steatosis and glucose tolerance, to increase metabolic rate, to decrease body fat mass, to reduce feeding, and to increase energy expenditure [35]. Moreover, the GLP-1/glucagon dual molecule induced body weight reduction and improved glycemia in non-human primates [34,117] and increased leptin sensitivity of DIO mice [37]. Interestingly, several peptides with different degrees of co-agonism for the glucagon and GLP-1 receptors were recently discovered [35], and are currently in clinical evaluation for the treatment of obesity and diabetes [115,118].

Another developed conjugate combines GLP-1 and amylin agonisms. Amylin is a pancreatic peptide co-secreted with insulin that induces hypoglycemic effects. Its levels have been shown to be decreased in type 1 diabetes [119]. Amylin has several physiological properties, such as regulating of the energy balance modulating the release of insulin, decreasing gastric acid secretion, delaying gastric emptying and inhibiting glucagon secretion [120,121,122]. Interestingly, central or peripheral amylin administration inhibits feeding, leading to body weight loss [121,123]. Thus, unimolecular peptide hybrids simultaneously targeting the receptors of GLP-1 and amylin induce body weight loss in obese rodent models with a higher efficiency than when administrated separately [124,125].

Another dual effect molecule concurrently targeting GLP-1 receptor and gastric inhibitory peptide (GIP) has been developed [126]. GIP is produced by K cells of the duodenum and jejunum, and is released in the circulation when lipids reach the intestinal tract. GIP exerts an insulinotropic effect, favoring pancreatic insulin release in humans [127], and recently it has been described that GIP could also stimulate glucagon release during hypoglycemia, acting as a buffer to counter the extreme changes in circulating glucose levels [128,129]. Moreover, the chronic activation of GIP receptor (GIPR) improves glucose metabolism in obese mice [130]. The efficiency of this single molecule combining the pharmacologic effects of GIP and GLP-1 is mainly due to the insulinotropic action of both compounds through different mechanisms [116,126], displaying higher effects on the reduction of body weight and on the improvement of both insulin resistance and glucose intolerance than when administrated separately [126].

Based on the beneficial effect of this elegant multi-agonism strategy, the next step was to target three receptors simultaneously. Thus, a unique molecule displaying the lipolytic effects of glucagon, the thermogenic properties of GLP-1, and the insulinotropic action of GIP has recently been developed. This conjugate has shown a significant effect on decreasing body weight through an increase of energy expenditure, together with an inhibition of feeding and improving hyperglycemia [131]. Interestingly, the beneficial effects of this tri-agonist have been observed in in both male and female rodents and non-human primates [34,132].

Finally, glucagon or GLP-1 receptor agonists have been combined with hormone nuclear receptor agonists for estrogens, androgens, and thyroid hormones, as the activation of their nuclear receptors is known to induce beneficial effects for metabolic syndrome. However, this strategy implies that the co-agonist molecules have to cross the plasma membrane to exert their hormonal actions. Interestingly, the GLP-1/estrogen conjugate has shown a higher efficiency for decreasing body weight and improving glycemic control in obese mice than when administrated individually [133]. Furthermore, glucagon and thyroid hormone effects have been combined in a conjugate that corrected dyslipidemia in several diet-induced obese rodent models, demonstrating their lipid and weight lowering effects, being partially dependent on increased thermogenesis [134].

Ultimately, anti-inflammatory effects of glucocorticoids have also been combined with GLP-1 agonism. Thus, a GLP-1/dexamethasone chimera synergistically improved body weight, glucose tolerance and insulin sensitivity in peripherally and centrally administrated obese mice [135].

## 5. Conclusions

Data gleaned over the last few years have allowed the unmasking of some of the mechanisms exerted by GLP-1 and/or its longer acting agonists in energy and metabolic homeostasis. In addition to their well-known beneficial effect on glycaemic control in type II diabetes by decreasing insulin resistance, their recently discovered lipid-lowering effects have further increased clinical expectations, particularly for the treatment of obesity [63,111,115]. This positive effect is likely due to a combination of several mechanisms. Firstly, their combined anorectic effects at a hypothalamic level and on slowing gastric emptying signals a limited substrate availability at a whole body level. On the other hand, their effect on BAT energy expenditure, and possibly on WAT browning or hepatic lipid oxidation, will lead to a depletion in endogenous lipid stores [87,97]. However, there are still a few outstanding questions concerning the underlying mechanisms, including a detailed assessment of the gastrointestinal vagal (and probably spinal) afferences mediating GLP-1 effects at a central level to elucidate the functions of each subset of neurons associated with specific regulatory process. This is particularly important since some effects exerted through this neuronal pathway have been reported for GLP-1 in glucose, lipid, and energy homeostasis. Additionally, although the role of BAT and browning in relation to energy expenditure has been studied in detail, the involvement of UCP1-independent mechanisms, as well as the putative existence of a new adipocyte population, should be further investigated.

The growing rate of obesity and diabetes in developed countries has aroused the concern of the scientific and pharmaceutical communities and the drive to seek effective treatments. Since the beginning of the 20th century, several drugs have failed clinically for the treatment of obesity. For instance, thyroid hormones strongly induce thermogenesis and increase metabolic rate [98,100,111,136,137,138] and amphetamines are potent appetite suppressors [111,139,140,141,142]. Serotonergic targets have also been studied to reduce food intake [143]. However, these potential treatments were discarded due to safety concerns such as deleterious cardiovascular effects. Despite its beneficial effect on body weight and metabolic parameters, the lipase inhibitor Orlistat also produces deficiencies in liposoluble vitamins [144,145]. Despite all these pharmacological approaches, the only effective, durable, and safe treatment against obesity is actually bariatric surgery [111]. However, in the last years, new, promising therapeutic strategies have emerged, mostly focused on the agonism of GLP-1 due to its demonstrated ability to reduce body weight and comorbidities. Interestingly, the use of GLP-1-agonists has minimized the serious adverse effects induced by the other treatments, despite the fact that the efficient dose needed to achieve its weight lowering effect is much greater than that needed to treat glucose homeostasis. The release of these new GLP1-based chimeras is expected to result in higher clinical efficacy with lower adverse effects for the treatment of obesity, and, likely, its pharmacological action will be extended to other comorbidities such as type II diabetes, nephropathy, and non-alcoholic fatty liver disease or steatohepatitis [34,35,111,126,132,134,135].
